# Iatrogenic Spinal Cord Herniation: Systematic Review of a Rare Postoperative Complication

**DOI:** 10.51894/001c.143749

**Published:** 2025-09-30

**Authors:** Dishaben Patel, Kelly Mei, Elise Yoon, Daniel Carr

**Affiliations:** 1 College of Osteopathic Medicine Michigan State University; 2 College of Osteopathic Medicine Michigan State University; 3 Neurosurgery Henry Ford Providence

## 35

### INTRODUCTION

Postoperative spinal cord herniation is a rare complication that may occur after spinal surgery for degenerative or neoplastic conditions in various spinal regions. Its mechanism is poorly understood.

### OBJECTIVE

We sought to characterize patients with iatrogenic spinal cord herniations and identify any potential risk factors.

### METHODS

A search of PubMed was conducted to perform a systematic review of the literature in accordance with Preferred Reporting Items for Systematic Reviews and Meta-Analysis (PRISMA) guidelines. Cases describing idiopathic or post-traumatic cord herniations and non-English publications were excluded.

### RESULTS

Thirty-one articles describing 31 patients were included in the study. Mean age was 47.19 ± 13.97 years, with a range of 15-74 years. The majority of patients were male (n=23, 74.2%). Index surgery was located dorsally in 71%, ventrally in 22.6%, and laterally in 6.4% of patients. There was a known durotomy in 77.4% of index surgeries while only 35.5% had an intended durotomy at the time of index surgery. Two patients had cord herniations after radiation therapy to the surgical site. Time from index surgery to symptomatic presentation ranged from 0 to 180 months, with a mean length of 48.78 ± 59.15 months.

Spinal cord herniations were most common in the cervical spine (n=21, 67.7%), followed by thoracic (n=8, 25.8%). Cord herniations spanned a mean of 1.55 ± 0.57 vertebral body levels. Almost all (96.8%) patients were symptomatic, and the most common symptom was weakness (90.3%), followed by sensory changes (61.3%). All but 3 patients (n=28, 90.3%) underwent surgery for repair of cord herniation. All patients who underwent surgical repair had improvement of their symptoms. Mean follow-up time was 48.78 ± 59.15 months.

### CONCLUSIONS

Patients who had iatrogenic spinal cord herniation were more likely to be male and have a dorsal index surgery with durotomy. Patients who underwent surgical repair were likely to have clinical improvement.

